# Fingolimod treatment modulates PPARγ and CD36 gene expression in women with multiple sclerosis

**DOI:** 10.3389/fnmol.2022.1077381

**Published:** 2022-12-15

**Authors:** Véronique Ferret-Sena, Carlos Capela, Ana Macedo, António Vasco Salgado, Bruno Derudas, Bart Staels, Armando Sena

**Affiliations:** ^1^Centro de Investigação Interdisciplinar Egas Moniz (CiiEM), Instituto Universitário Egas Moniz, Monte de Caparica, Portugal; ^2^Departamento de Neurologia, Hospital Santo António dos Capuchos, Centro Hospitalar Universitário de Lisboa Central, Lisbon, Portugal; ^3^Keypoint Consultora Científica, Algés, Portugal; ^4^Departamento de Ciências Biomédicas e Medicina (DCBM), Universidade do Algarve, Faro, Portugal; ^5^Serviço de Neurologia, Hospital Fernando da Fonseca, Amadora, Portugal; ^6^Inserm, CHU Lille, Institut Pasteur de Lille, University of Lille, Lille, France

**Keywords:** fingolimod, peroxisome proliferator-activated receptors (PPAR), cluster of differentiation 36 (CD36), lipoproteins, multiple sclerosis

## Abstract

Fingolimod is an oral immunomodulatory drug used in the treatment of multiple sclerosis (MS) that may change lipid metabolism. Peroxisome proliferator-activated receptors (PPAR) are transcription factors that regulate lipoprotein metabolism and immune functions and have been implicated in the pathophysiology of MS. CD36 is a scavenger receptor whose transcription is PPAR regulated. The objective of this study was to evaluate whether fingolimod treatment modifies PPAR and CD36 gene expression as part of its action mechanisms. Serum lipoprotein profiles and PPAR and CD36 gene expression levels in peripheral leukocytes were analysed in 17 female MS patients before and at 6 and 12 months after fingolimod treatment initiation. Clinical data during the follow-up period of treatment were obtained. We found that fingolimod treatment increased HDL-Cholesterol and Apolipoprotein E levels and leukocyte PPARγ and CD36 gene expression. No correlations were found between lipid levels and variations in PPARγ and CD36 gene expression. PPARγ and CD36 variations were significantly correlated during therapy and in patients free of relapse and stable disease. Our results suggest that PPARγ and CD36-mediated processes may contribute to the mechanisms of action of fingolimod in MS. Further studies are required to explore the relation of the PPARγ/CD36 pathway to the clinical efficacy of the drug and its involvement in the pathogenesis of the disease.

## Introduction

Fingolimod (FTY720, Gilenya) was the first oral disease-modifying treatment (DMT) approved in MS. Fingolimod is a synthetic sphingosine analogue which once phosphorylated to sphingosine-1-phosphate (S1P) binds to several S1P receptors (S1PR). High levels of S1PR1 receptor subtype are expressed in lymphocytes and required for their egress from lymphoid organs. By inducing internalization and degradation of S1PR1, phospho-FTY720 impairs this egress, resulting in a significant reduction of circulating T and B cells and infiltration in the CNS (Roy et al., 2021). Modulation of S1P receptors present in endothelial cells, neurons, glial cells and the innate immune system may also contribute to the efficacy S1PR-directed therapies in MS (Thomas et al., 2017; Roy et al., 2021; Mirzaei et al., 2022). Within circulation, S1P is mainly present in high-density lipoprotein (HDL-C), and mediates the regulatory properties of this lipoprotein in immune responses (Blaho et al., 2015). Interestingly, it was recently observed that fingolimod increases plasma HDL-Cholesterol (HDL-C) levels in MS patients, although no association with the anti-inflammatory effects of the treatment was found (Blumenfeld Kan et al., 2019). Dyslipidaemia has been suggested to play a role in MS pathogenesis and some studies have shown the protective effects of HDL-C on disability progression and development of new brain lesions in MS patients (Zhornitsky et al., 2016). However, as in other chronic inflammatory disorders, the profile and composition of lipoprotein sub-fractions could be altered in MS patients and result in a dysfunctional HDL with impaired anti-inflammatory effects (Jorissen et al., 2017). In sum, the molecular mechanisms underlying the therapeutic effects of fingolimod in MS are complex and not fully understood. Here, we questioned whether peroxisome proliferator-activated receptors (PPAR) could be implicated in the mechanism of action of fingolimod therapy in these patients.

PPARs are ligand-activated transcriptional factors involved in the regulation of lipid and glucose metabolism and adaptive and innate immunity. The PPAR subfamily of nuclear receptors comprises the members PPARα (NR1C1), PPARβ/δ (NR1C2) and PPARγ (NR1C3) which, after ligand activation, regulate gene transcription by dimerizing with the retinoid X receptor and acting on specific DNA sequences. PPAR can regulate gene expression also by interfering with other transcriptional factors and other proteins implicated in human disease (Rigamonti et al., 2008; Surgucheva and Surguchov, 2008). PPAR are widely distributed in human cells, including in the CNS, and have been implicated in the pathophysiology of MS (Ferret-Sena et al., 2018). For instance, PPARγ expression in CNS myeloid cells was shown to control inflammatory activation in experimental autoimmune encephalomyelitis (EAE) (Hucke et al., 2012) and its agonist pioglitazone decreases systemic inflammatory activity and the development of new brain lesions in MS obese patients (Negrotto et al., 2016). In line with this scenario, recent work has shown that MS-associated inflammatory mediators reduce the expression of PPARγ in monocyte-derived macrophages from healthy subjects (Wouters et al., 2020). A significant amount of data supports a major role of PPAR in the cross-talk between lipoprotein metabolism and inflammatory responses (Rigamonti et al., 2008; Ferret-Sena et al., 2018). In low HDL-C subjects, increased expression of inflammatory genes in monocyte-derived macrophages is associated with decreased expression of PPARγ (Sarov-Blat et al., 2007) and pioglitazone treatment increases HDL-C levels (Kabeya et al., 2011). In addition, PPARγ agonists increase S1PR1 expression in lymphocytes and regulate intracellular and blood levels of S1P (Liu et al., 2016; Kurano et al., 2018). Cluster of Differentiation 36 (CD36) is a membrane receptor upregulated by PPARγ expressed in many cells that modulates immune functions (Rigamonti et al., 2008; Silverstein and Febbraio, 2009). CD36 is a scavenger receptor for oxidized low-density lipoprotein (oxLDL) and other lipids generated by inflammatory processes during atherogenesis (Silverstein and Febbraio, 2009) and implicated in reparative mechanisms of MS lesions (Grajchen et al., 2018, 2020). Based on these data, the present exploratory study investigated PPAR and CD36 gene expression in blood leukocytes and associated plasma lipoprotein profile in MS patients under fingolimod treatment.

### Materials and methods

#### Patients enrolment

Seventeen female patients with relapsing–remitting MS (RRMS) (Polman et al., 2005) were recruited from two MS clinical centres in Lisbon (Portugal). All individuals who received fingolimod as a second-line therapy (0.5 mg/day) due to prior failure of disease-modifying treatment (DMT) were studied during a follow-up period of 12 months. The Expanded Disability Status Score (EDSS) (Kurtzke, 1983) was determined at the enrolment time and after 6 months and 12 months of treatment. Number of relapses in the last year previously to treatment and during the follow-up period was also recorded. Relapses were defined as the appearance or worsening of neurological signs lasting over 24 h and not associated with fever. Clinical indexed information included age at disease onset and disease duration, previous therapies, body mass index (BMI) (kg/m^2^), current smoking habits and oral contraceptive use. Data on magnetic resonance imaging (MRI) and blood cell counts from the 1-year follow-up were also collected. Brain MRI at 1.5 Tesla with and without gadolinium infusion was performed according to the standard procedures. No evidence for disease activity (NEDA-3) was defined by the absence of relapses and sustained EDSS progression and no new or enlarging T2-weighted and contrast-enhancing lesions on T1-weighted. None of the patients were taking lipid-lowering medication before and during the study or suffered from any metabolic disorder. All patients signed informed consent and the study was conducted according to the Declaration of Helsinki and approved by the local Ethics Committees (Central Lisbon University Hospital Centre and Fernando da Fonseca Hospital).

#### Blood collection, isolation of leukocytes, and RNA extraction

Blood samples were collected by venepuncture in fasting conditions and coded for blinded analysis. Purification of mRNA from leukocytes was performed according to QIAamp RNA Blood Mini Kit (Qiagen) protocol. Erythrocytes were selectively lysed and leucocytes recovered by centrifugation, processed immediately and RNA stored at-80°C until analysis. Plasma sample was also collected and stored at-80°C until use for lipid and apolipoprotein analysis.

#### Lipid and apolipoprotein analysis

Serum triglycerides (TG), total cholesterol (TC), high-density lipoprotein (HDL), and low-density lipoprotein (LDL) cholesterol (HDL-C and LDL-C) were measured enzymatically by using the Roche automated clinical chemistry analyser (COBAS 6000). Non-HDL-C cholesterol levels were calculated by subtracting HDL-C from TC. Apolipoprotein A-1 (ApoA1), Apolipoprotein B (ApoB), and Apolipoprotein E (ApoE) were determined by turbidimetry methods (Roche, COBAS 6000). Lipoprotein(a) (Lp(a)) was measured by nephelometry. Oxidized LDL (oxLDL) was determined by commercially available Enzyme-Linked Immunosorbent Assay (ELISA) kits from Mercodia eBioscience.

#### Quantitative PCR

PPARα, PPARβ/δ, PPARγ, and CD36 mRNA expression in leucocytes was evaluated by quantitative RT-PCR. RNA was reverse-transcribed using random hexamer primers and Superscript reverse transcriptase (Life Technologies, France) and cDNAs were quantified using specific oligonucleotides (for PPAR, CD36, and cyclophilin) by Master Mix II Agilent on an Mx3000 apparatus Stratagene, La Jolla, CA (see Supplementary Information for primers and probes used). The relative expression of each gene was calculated by the ΔCt method, where ΔCt is the value obtained by subtracting the Ct (threshold cycle) value of cyclophilin mRNA from the Ct value of the target gene. The amount of target relative to the cyclophilin mRNA was expressed as 2^−(ΔCt)^.

#### Statistical analysis

Statistical analysis was performed by an expert statistician using SPSS version 21.0 and R version 3.6.1. Variations during the 12-month follow-up were assessed by Friedman test. Data from the 6-month and 12-month follow-ups were compared with baseline data through a nonparametric Wilcoxon test. The correlations between the changes in HDL-C, PPARγ and CD36 and other variables were carried out using Spearman’s rank correlation coefficient. Fold changes were estimated for this purpose to quantify changes between baseline and 12 months and obtained by the ratio of the two quantities. A value of *p* < 0.05 was considered statistically significant.

## Results

The main demographic and clinical characteristics of the 17 MS female patients treated with fingolimod are summarized in Table 1. All patients received fingolimod as a second-line therapy. Interferon β was the most frequent previous DMT (76.5%). Mean body mass index (BMI) was stable in most patients. A decrease in BMI by more than one point was observed in 2 patients (12%). EDSS was stable and the mean relapse rate of the patient cohort in comparison to the last year prior to treatment significantly decreased (*p* < 0.001). Five patients remained with clinical and/or imaging evidence of disease activity. As expected, a significant decrease in absolute lymphocyte count (ALC) and an increase in the neutrophil/lymphocyte ratio during fingolimod treatment were observed (*p* = 0.001). Eleven patients were hormonal contraceptive users and five patients were smokers since treatment initiation.

**Table 1:** Patient’s demographic and clinical data.

	Baseline	12 months	*p*-Value
Female, n (%)	17 (100)	–	–
Age, years	39.1 ± 8.5	–	–
Disease duration, years	9.2 ± 6.9	–	–
Previous DMT Interferon β, *n* (%)	13 (76.5)	–	–
BMI, kg/m^2^	24.5 ± 5.1	24.7 ± 5.7	0.708
EDSS	2.6 ± 2.2	2.8 ± 2.1	0.867
ARR	1.4 ± 0.9	0.1 ± 0.3	**<0.001**
Lymphocytes, × 10^9^/L	1.7 ± 0.9	0.6 ± 0.5	**0.001**
N/L ratio	3.6 ± 3.1	7.7 ± 4.3	**0.001**

DMT, disease-modifying therapy; BMI, Body Mass Index; EDSS, Expanded Disability Status Scale; ARR, annualized relapse rate; N/L, neutrophils/lymphocytes. Data are presented as mean and standard deviation (SD), value of ps obtained through nonparametric Wilcoxon test. Bold values means significant differences.

Table 2 summarizes patients lipid profile assessed at baseline, 6-months, and 12-months after fingolimod initiation. At baseline, 17.6% of patients had HDL-C at the recommended level (≥60 mg/dl), but this percentage increased to 29.4% at 6 and 12 months of follow-up. This treatment was associated with a significant increase in HDL-C (*p* = 0.015), with a 10.4% increase after 6 months (*p* = 0.014) and 15.7% increase after 12 months (*p* = 0.002). No significant differences for other lipids and lipid ratios were found in patient’s post-treatment compared with pre-treatment levels. Regarding apolipoproteins, a significant increase in ApoE was found at 12 months of treatment (*p* = 0.049). No correlation was found between HDL-C levels and ALCs.

**Table 2:** Patient’s lipid profile.

	Baseline	6 months	12 months	*p*-Value (*)
TC (mg/dl)	156.1 ± 29.7	160.2 ± 30.7	168.4 ± 27.8	0.056
HDL-C (mg/dl)	48.9 ± 11.0	54.0 ± 12.4	56.6 ± 15.6	**0.015**
Non-HDL-C (mg/dl)	107.2 ± 36.2	106.2 ± 32.9	111.8 ± 29.9	0.257
TC/HDL-C	3.4 ± 1.4	3.1 ± 1.0	3.2 ± 0.9	0.327
LDL-C (mg/dl)	85.7 ± 29.9	87.7 ± 30.2	91.8 ± 23.8	0.073
TG (mg/dl)	106.8 ± 61.8	92.6 ± 36.3	99.9 ± 45.1	0.146
ApoA1 (mg/dl)	149.6 ± 23.1	157.3 ± 24.1	156.3 ± 30.9	0.191
ApoB (mg/dl)	77.8 ± 27.3	75.8 ± 24.3	80.4 ± 19.2	0.280
ApoE (mg/dl)	3.0 ± 0.9	3.2 ± 1.1	3.5 ± 0.8	**0.049**
ApoB/A1 ratio	0.54 ± 0.22	0.49 ± 0.19	0.54 ± 0.18	0.838
Lp(a) (mg/dl)	19.6 ± 19.2	21.5 ± 22.7	24.1 ± 23.4	0.193
Oxidized LDL (mg/dl)	38.9 ± 16.4	45.3 ± 15.2	50.9 ± 21.9	0.627
OxLDL/LDL ratio	19.1 ± 8.4	21.9 ± 11.1	21.8 ± 9.2	0.420

^*^Comparisons between Baseline and 12 months. TC, total cholesterol; HDL-C, high-density lipoprotein cholesterol; LDL-C, low-density lipoprotein; cholesterol; TG, Triglycerides; ApoA1, Apolipoprotein A1; ApoB, Apolipoprotein B; ApoE, Apolipoprotein E; Lp(a), Lipoprotein(a). Data are presented as mean and standard deviation (SD). Value of ps obtained through nonparametric Wilcoxon test. Bold values means significant differences.

**Figure 1:**
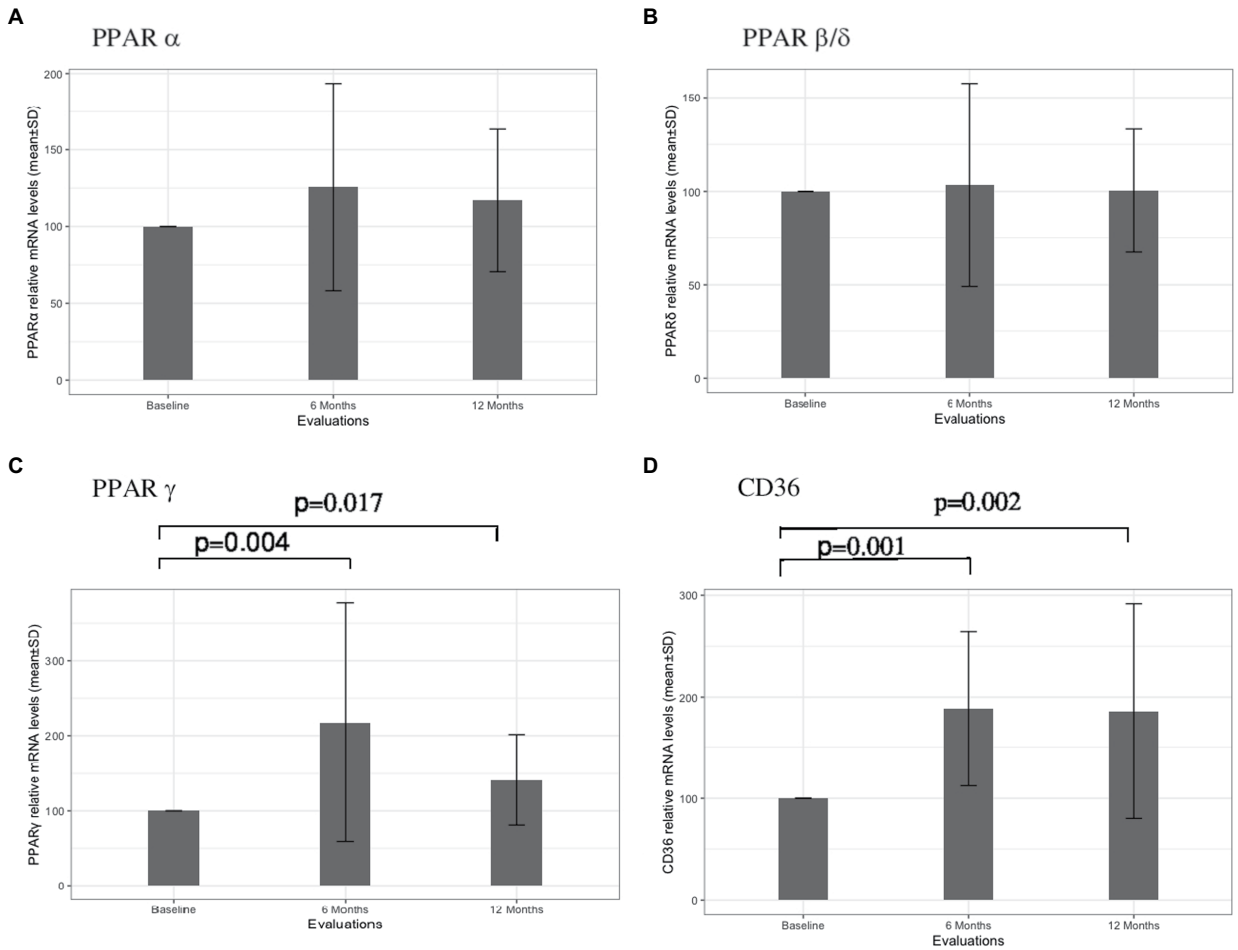
PPARα **(A)**, PPARβ/δ **(B)**, PPARγ **(C)**, and CD36 **(D)** leukocyte gene expressions at baseline, and at 6 and 12 months of treatment with Fingolimod. Value of ps obtained through nonparametric Wilcoxon test are presented.

**Figure 2:**
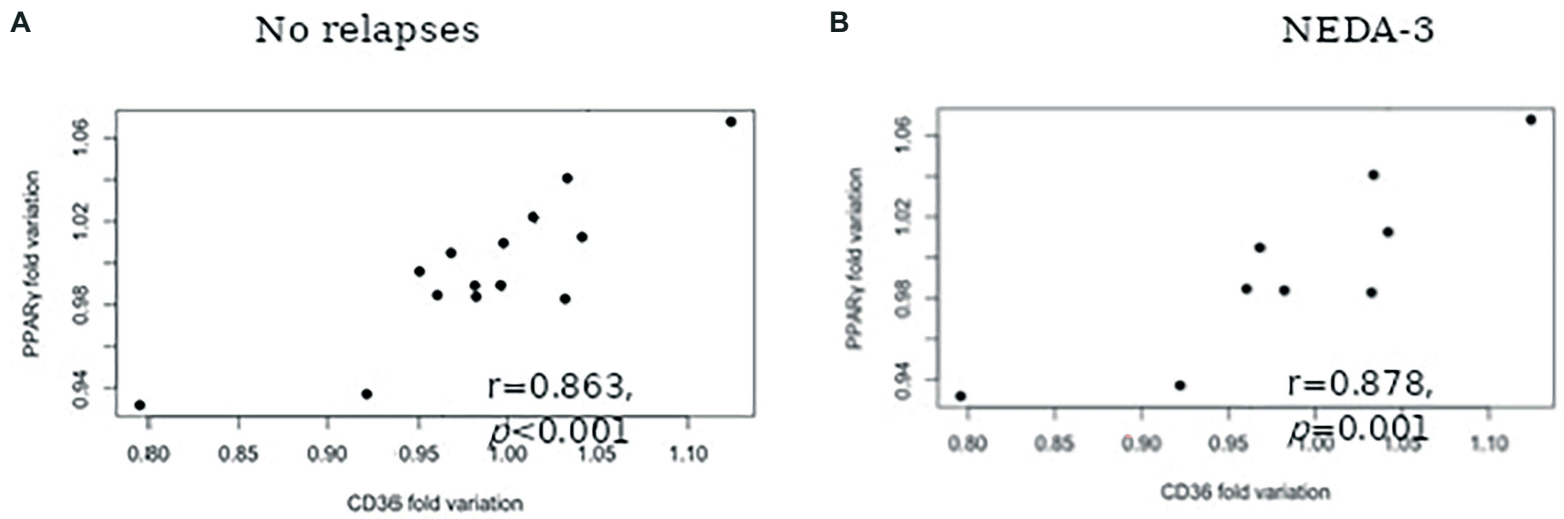
Spearman correlation between PPARγ fold variation and CD36 fold variation for patients with: No relapses during the 12-months follow-up **(A)** and No evidence of disease activity (NEDA-3) **(B)**.

The results concerning PPARS and CD36 mRNA expression in blood leukocytes are presented in Figure 1. No significant alterations in PPARα (Figure 1A) and PPARβ/δ (Figure 1B) gene expression were found at 6 and 12 months after therapy. At 6 months, patients had higher PPARγ mRNA expression in comparison to the baseline (mean difference 117.9, *p* = 0.004). Although a slight decrease in the relative mRNA levels was observed at 12 months, PPARγ mRNA expression remained higher in comparison to the baseline (mean difference 41.1, *p* = 0.017) (Figure 1C). Higher CD36 gene expression was also observed at 6 months in comparison to baseline (mean difference 88.4, *p* = 0.001). Similar mRNA levels were found at 12 months, with a mean difference of 85.9 to baseline levels (*p* = 0.002) (Figure 1D). A significant correlation between PPARγ and CD36 was observed for the cohort of patients at six (*r* = 0.549, *p* = 0.022) and 12 (*r* = 0.748, p = 0.001) months of treatment but not at baseline (*r* = 0.463, *p* = 0.061). Furthermore, a strong correlation between PPARγ variation and CD36 variation was found for patients with no relapses (*r* = 0.863, *p* < 0,001) (Figure 2A) and stable disease activity (*r* = 0.878, *p* = 0.001) during treatment (Figure 2B). No significant correlations were observed between PPARγ and CD36 variations and HDL-C levels and ALCs.

## Discussion

Dyslipidaemia has been implicated in the pathogenesis of the MS (Zhornitsky et al., 2016) and fingolimod therapy was recently observed to change the serum lipid profile in these patients (Blumenfeld Kan et al., 2019). In agreement with this last study, we found specific increases of HDL-C levels at 6 and 12 months after treatment initiation. As also reported by Blumenfeld Kan et al. (2019) (Blumenfeld Kan et al., 2019), fingolimod therapy was not associated with significant changes in BMI, despite an increase of the percentage of patients reaching the recommended level of HDL-C ≥ 60 mg/dl. Some studies have shown that higher levels of HDL-C are associated with anti-inflammatory effects in MS (Weinstock-Guttman et al., 2013; Fellows et al., 2015). In contrast to fingolimod, natalizumab therapy in MS is linked to sequestration of activated lymphocytes in the systemic circulation and decreases plasma HDL-C levels (Moccia et al., 2018). However, as previously observed (Blumenfeld Kan et al., 2019), no correlation between HDL-C levels and absolute lymphocyte counts (ALCs) was found in the present study. Whether the increase in HDL-C induced by fingolimod may represent a potential biomarker of the protective effects of the drug remains to be explored. Beyond prominent effects on lymphocyte trafficking (Roy et al., 2021), fingolimod has slower effects on innate immune cells (Thomas et al., 2017). In this context, it is intriguing that an increase of ApoE levels was only observed after 1-year of therapy, suggesting a slower alteration of apolipoprotein metabolism by the drug. A potential role of ApoE in the pathophysiology of MS is equivocal (Zhornitsky et al., 2016). However, recent studies suggest an involvement of ApoE in modulating disability progression (Sena et al., 2019; McComb et al., 2020). Moreover, myeloid-derived ApoE present in HDL-C controls the innate and adaptive immune reactivities and its deficiency may promote autoimmune responses (Bonacina et al., 2018). Longer follow-up studies are warranted to characterize the composition of lipoprotein fractions in fingolimod-treated patients and its potential contribution to the mechanisms of action of the drug.

The main objective of the current study was to investigate whether PPAR could be involved in the mechanisms of action of fingolimod in MS patients. PPAR have been implicated in the pathophysiology of EAE and MS and are major players in the interactions between inflammatory responses and lipid metabolism (Ferret-Sena et al., 2018). Specific increases of PPARγ and CD36 gene expressions in blood leukocytes were observed during the one-year follow-up period of the study. It is well known that PPAR repress the expression of several proinflammatory mediators that are involved in MS pathogenesis. MS patients exhibit decreased PPARγ expression in peripheral blood mononuclear cells (PMNC) compared with controls (Klotz et al., 2005) and in obese MS patients, pioglitazone (a PPARγ agonist) decreases inflammatory activity and the development of new brain lesions (Negrotto et al., 2016). Furthermore, PPARγ expression in CNS myeloid cells was shown to control inflammatory activation in EAE (Hucke et al., 2012). Despite the potential involvement of other PPAR subtypes in the disease, it is remarkable that only PPARγ protein concentration in CSF was found to be increased in these patients, possibly reflecting a protective mechanism to counteract the inflammatory activity of the disease (Szalardy et al., 2017). Supporting this hypothesis, recent work has shown that MS-associated inflammatory mediators reduce PPARγ mRNA expression in monocyte-derived macrophages from healthy donors (Wouters et al., 2020). These data suggest that the specific PPARγ gene expression induced by fingolimod may be associated with the anti-inflammatory effects of the drug. Interestingly, there is evidence for physiological interactions between PPARγ and S1P. PPARγ directly interacts with S1P (Parham et al., 2015) and PPARγ activation increases S1P synthesis and upregulates S1PR expression (Liu et al., 2016; Kurano et al., 2018). Induction of S1PR1 expression by PPARγ affects immune cells trafficking, macrophage and T cell polarization and B-cells activation (Liu et al., 2016). Fingolimod has protective effects on blood–brain barrier (BBB) permeability and leukocyte migration by blocking the expression and activation of multiple signaling molecules in endothelial cells (Zhao et al., 2018). In several experimental models, PPARγ activity has been shown to promote BBB integrity (Sivandzade and Cucullo, 2019). PPARγ activation is an important signal for CD36 expression, a receptor involved in inflammatory and oxidative stress regulation (Rigamonti et al., 2008; Silverstein and Febbraio, 2009; Sini et al., 2017). Increased CD36 expression in macrophages/microglia may play crucial roles in suppression of inflammatory activity and in promoting repair mechanisms of MS lesion (Grajchen et al., 2020). CD36 is a scavenger receptor for oxLDL and other oxidized lipids and is implicated in the phagocytosis of myelin debris in MS lesions (Grajchen et al., 2018). In MS, elevated levels of circulating oxLDL were associated with adverse clinical outcomes (Zhornitsky et al., 2016). Interestingly, fingolimod therapy was not associated with alterations in serum oxLDL levels and oxLDL/LDL ratio. In line with this scenario, we have reported that the systemic inflammatory activity induced by natalizumab therapy is associated with a reduction of PPARγ and CD36 gene expressions in PMNC (Ferret-Sena et al., 2016). Dysfunctional HDL, with proinflammatory propriety, may be present in MS (Jorissen et al., 2017)and could stimulate the PPARγ/CD36 pathway (Sini et al., 2017). However, no correlations between PPARγ and CD36 variations and HDL-C levels and ALCs were found. Moreover, PPARγ and CD36 gene expressions were not correlated at baseline, but significantly correlated during fingolimod treatment and in patients free of disease activity. It should be noted that no direct link between ALCs and therapeutic response to fingolimod has been observed, although this drug induces selective changes in blood lymphocytes subsets that were not assessed in this present study (Boffa et al., 2020). Since fingolimod is a lipophilic molecule, it crosses the BBB and may exert protective effects within the CNS by inducing metabolic reprogramming and neuroinflammatory modulation (Mirzaei et al., 2022). Taken together, our data suggest that activation of the PPARγ/CD36 pathway could contribute to the reparative and neuroprotective effects of fingolimod therapy in MS patients.

The main limitation of this study concerns the small cohort of participants, preventing comparison between patients with active versus stable disease. As only women were included, our results cannot be generalized to male patients because immune regulation by PPAR may depend on gender (Park and Choi, 2017). However, there is no evidence for gender-related differences in the mechanisms of action and clinical efficacy of fingolimod. Therefore, it seems likely that similar data may be anticipated for male patients. Although the use of oral contraceptives may alter the lipoprotein profile in MS patients (Sena et al., 2019), all women taking oral contraceptives started their use prior to fingolimod treatment and did not change its formulation. It seems unlikely that prior treatments could have biased the results, as the vast majority of patients were treated with interferon β formulations. Unfortunately, due to insufficient blood volume, PPARγ and CD36 protein levels could not be determined. In sum, additional studies in a larger cohort of participants including male gender and longer follow-up analyses are needed to confirm the pathophysiological and clinical relevance of the present results. Vitamin D levels were not assessed. Nevertheless, previous work found no correlation between the lipid profile and vitamin D levels in patients treated with fingolimod (Blumenfeld Kan et al., 2019). Data on changes in lifestyle, diet, and physical activity during the follow-up period were not collected. However, during treatment, body weight remained stable and an effect attributed to these factors seems unlikely.

In conclusion, this pilot study indicates that fingolimod therapy increases PPARγ and CD36 gene expressions in circulating leukocytes of women with MS. PPARγ and CD36 variations were correlated during treatment but not with associated changes in the serum lipoprotein profile. Further studies are required to assess the contribution of the PPARγ/CD36 pathway for the clinical efficacy of the drug and the pathogenesis of MS.

## Data availability statement

The original contributions presented in the study are included in the article/Supplementary material, further inquiries can be directed to the corresponding author.

## Ethics statement

The studies involving human participants were reviewed and approved by Ethic Committee of Central Lisbon University Hospital Centre Ethic Committee of Fernando da Fonseca Hospital. The patients/participants provided their written informed consent to participate in this study.

## Author contributions

AS and VF-S: conceptualization. BD, BS, and VF-S performed the laboratory analysis. CC and AS monitored patients. AM conducted the statistical analysis. AS wrote the first draft of the manuscript and all authors contributed to the manuscript revision, to data interpretation and approved the submitted version.

## Funding

This study received financial support from Novartis Farma Portugal and National Funds through the FCT-Foundation for Science and Technology, I.P, under the project UIDB/04585/2020.

## Conflict of interest

The authors declare that the research was conducted in the absence of any commercial or financial relationships that could be construed as a potential conflict of interest.

## Publisher’s note

All claims expressed in this article are solely those of the authors and do not necessarily represent those of their affiliated organizations, or those of the publisher, the editors and the reviewers. Any product that may be evaluated in this article, or claim that may be made by its manufacturer, is not guaranteed or endorsed by the publisher.
