# Supplementary material for: Fingolimod treatment modulates PPARγ and CD36 gene expression in women with multiple sclerosis
Source: Front Mol Neurosci. 2022 Dec 15;15:1077381. doi: 10.3389/fnmol.2022.1077381 (PMC9797671; doi:10.3389/fnmol.2022.1077381)
Supplement: Supplementary file 1 [file Table_1.DOCX]

**Supplementary Information on Primers and Probes used**

|  | Sens | Antisens |
| --- | --- | --- |
| Cyclophiline | GCA TAC GGG TCC TGG CAT CTT GTC C | ATG GTG ATC TTC TTG CTG GTC TTG C |
| PPARα | GGT GGA CAC GGA AAG CCC AC | GGA CCA CAG GAT AAG TCA CC |
| PPAR β/δ | GCA TGA AGC TGG AGT ACG AGA AG | GCA TCC GAC CAA AAC GGA TA |
| PPARγ | GAG CGG GTG AAG ACT CAT GTC TGT C | \| AGT CCT CAC AGC TGT TTG CCA AGC \| \| --- \| |
| CD36 | GGT TGA CCT GCA GCC GTT TTG | \| TCA GCA AAT GCA AAG AAG GGA GAC \| \| --- \| |
